# Impact of Farmland Change on Soybean Production Potential in Recent 40 Years: A Case Study in Western Jilin, China

**DOI:** 10.3390/ijerph15071522

**Published:** 2018-07-18

**Authors:** Luoman Pu, Shuwen Zhang, Fei Li, Ranghu Wang, Jiuchun Yang, Liping Chang

**Affiliations:** 1 College of Earth Science, Jilin University, Changchun 130012, China; puluoman@sina.com; 2 Northeast Institute of Geography and Agroecology, Chinese Academy of Sciences, Changchun 130102, China; yangjiuchun@iga.ac.cn (J.Y.); lpchang@iga.ac.cn (L.C.); 3 College of Unban and Environmental Science, Northwest University, Xi’an 710127, China; lifei@nwu.edu.cn; 4 Institute of Loess Plateau, Shanxi University, Taiyuan 030006, China; wangranghu@163.com

**Keywords:** farmland change, soybean, production potential, GAEZ, Western Jilin

## Abstract

During the last 40 years, the quantity and spatial patterns of farmland in Western Jilin have changed dramatically, which has had a great impact on soybean production potential. This study used one of the most advanced crop production potential models, the Global Agro-Ecological Zones model, to calculate the soybean production potential in Western Jilin based on meteorological, topography, soil and land use data, and analyzed the impact of farmland change on soybean production potential during 1975–2013. The main conclusions were the following: first, the total soybean production potential in Western Jilin in 2013 was 8.92 million tonnes, and the average soybean production potential was 1612 kg/ha. The production potential of eastern area was higher than the other areas of Western Jilin. Second, farmland change led to a growth of 3.30 million tonnes in soybean production potential between 1975 and 2000, and a decrease of 1.03 million tonnes between 2000 and 2013. Third, taking account of two situations of farmland change, the conversion between dryland and other categories, and the change of irrigation percentage led to the total soybean production potential in Western Jilin increased by 2.31 and only 0.28 million tonnes respectively between 1975 and 2000, and increased by 0.12 and 0.29 million tonnes respectively between 2000 and 2013. In general, the increase of soybean potential production was mainly due to grassland and woodland reclamation. The results of this study would be a good guideline for protecting safe baseline of farmland, managing land resources, and ensuring continuity and stability of soybean supply and food security.

## 1. Introduction

Food is a specialized commodity and an important strategic reserve relating directly to a country’s well-being [1,2]. Food security is an important part of national security. Soybean is one of the most important food and oil crops in China. Soybean imports in China increased nearly 100 times, from 580 thousand tonnes in 1996 to 58.38 million tonnes in 2012, while soybean production has been around 15 million tonnes and even has started to decline in recent years [3]. Recently, China’s agricultural administration has called for a restructuring of agricultural production to increase the soybean planting area. Therefore, to improve soybean yield and ensure soybean supply and guide import and export, it is of great significance to study soybean production potential. 

At present, research on crop production potential is extensive. Many scholars have explored new methods to study crop production potential as close as possible to the present state of land productivity. In 1950, Ren discussed land carrying capacity based on agricultural productivity, as the beginning of the study on China’s food production potential [4]. In 1978, Dutch scientist Wit et al. developed EL-CROS (Elementary Crop Growth Simulator) model, the first crop computational simulation model [5]. Meanwhile, the influence of climate factors such as temperature and precipitation began to be taken seriously. Some scholars used related functions to modify crop photosynthetic potential and gradually to the study phase of climate production potential [6,7,8]. Since 2000, many food production models have been established, such as the CERES (Crop Estimation through Resource and Environment Synthesis) wheat model [9], EPIC (Erosion/Productivity Impact Calculator) model [10], and WOFOST (World Food Studies) model [11]. However, the application of the Agro-Ecological Zones (AEZ) model has been the most extensive. The AEZ model was developed by the International Institute for Applied Systems Analysis (IIASA) and the Food and Agriculture Organization of the United Nations (FAO), and it calculated the food production potential under the influence of light, temperature, water, soil and topography [12,13,14,15]. Cai et al. analyzed China’s farming, and calculated wheat and rape yield potential using the AEZ model [16,17]. Yu et al. calculated maize production potential in Gansu Province using the AEZ model and predicted that maize production potential would 6072.67 kg/hm^2^ by 2015 [18]. Zhan et al. improved the AEZ model and studied dynamics change of the grain productivity in China [19]. Pan et al. explored the land use change in Taihu Lake Basin and the impact on cultivated land productivity, and found that although low-yield farmland increased, the decrease of high-yield farmland still resulted in the reduction of cultivated land productivity [20]. Alan et al. analyzed the effects of Agro-Ecological Zones and land use region boundaries on land resource projection using the Global Change Assessment Model [21]. Rachidatou et al. analyzed the distribution, pathological and biochemical characterization of *Ralstonia solanacearum* in Benin using AEZ model [22]. Nazrul et al. studied production potential and economics of mung bean in rice based cropping pattern in Sylhet region by using AEZ model [23]. Wang et al. studied the spatial-temporal characteristics of winter wheat yield gaps in Henan [24]. Although the research methods of crop production potential are various, one of the most mature models is the Global Agro-Ecological Zones (GAEZ) model developed from the AEZ model. The GAEZ comprehensively considers the radiation, temperature and other climatic factors that affect the crop growth, such as the length of the growing season, the water needs in different growth stages, etc. [13]. The current GAEZ (GAEZ v3.0) provides a major update of data and extension of the methodology compared to the earlier version [12]. The highlight of this study was using the most advanced GAEZ model to predict soybean production potential of Western Jilin in recent 40 years.

Western Jilin, located on the edge of a farming-pastoral zone in Northern China, is one of three saline-alkali landscapes in the world with soil that severely restricts its use for farming. Soybean is one of the common crops in Western Jilin. Thus, its farmland change would directly have a significant impact on soybean production potential [2]. Therefore, the objective of this study is to use the Global Agro-Ecological Zones (GAEZ) model to estimate the soybean production potential based on farmland data from 1975, 2000 and 2013, soil data, Digital Elevation model (DEM) data, and meteorological data from the past 40 years, and analyze the impact of farmland change on soybean production potential in Western Jilin. Under the background of global food security, the results of the research would provide a basis for the sustainable utilization of land resources and soybean production increase, provide a guideline for optimized allocation of land resources.

## 2. Materials and Methods

### 2.1. Study Area

**Figure 1:**
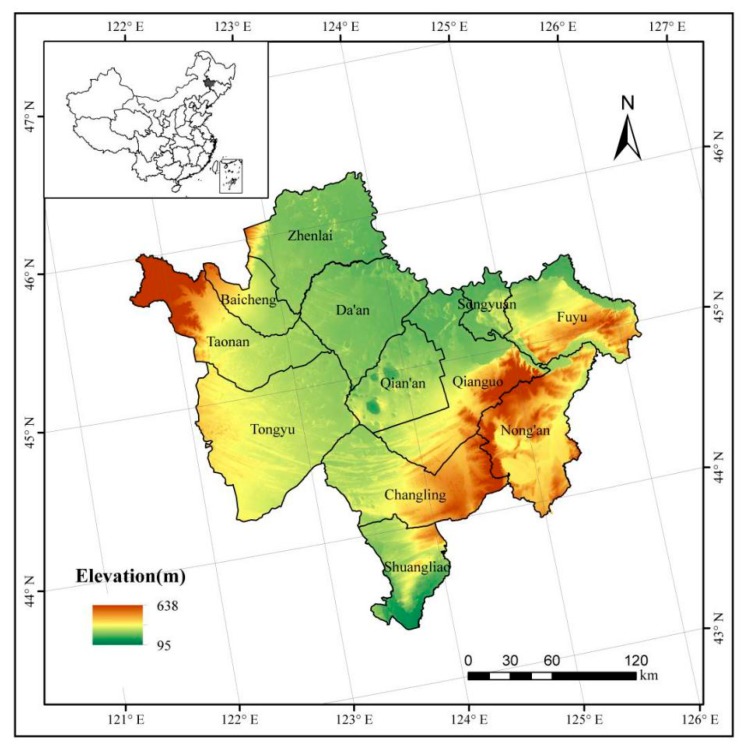
Location of Western Jilin, China.

Western Jilin Province is located in the southwest of the Songnen Plain, and is located at 43°22′—46°18′ N, 121°36′—126°12′ E. It includes 12 counties (or cities); Zhenlai, Baicheng, Taonan, Tongyu, Da’an, Qian’an, Songyuan, Fuyu, Qianguo, Songyuan, Changling and Shuangliao (Figure 1). The region is transitional from black soil in temperate sub-humid areas to chestnut soil in a temperate sub-arid steppe and typical farming-pastoral ecotone. The topography of Western Jilin is sloped from east and west to the middle [2,25]. The total area is about 5.53 million ha. The annual precipitation is between 370 and 410 mm, and ≥10 °C annual accumulated temperature is 2900–3200 °C. Western Jilin is mainly occupied by farmland, which covers 57.46% of the total area. Figure 1 shows the location of Western Jilin. The elevation data in the figure came from the DEM data with 90 m spatial resolution.

### 2.2. Data Source

Crop production potential within the region was strongly associated with various factors, such as farmland, soil, topography, and climate, so the input data for this study included land use data, topography elevation data, soil data, meteorological data and some statistical data.

The land use data of Western Jilin used in this study was extracted from the land-use database developed by the Chinese Academy of Sciences (CAS) (with a mapping scale of 1:100,000), in years 1975, 2000 and 2013. The land use database was obtained from manual visual interpretation at Landsat Thematic Mapper/Enhanced Thematic Mapper (TM/ETM) images. The land use data was classified into six major categories and 25 sub-categories. The six major categories included farmland, woodland, grassland, water bodies, built-up land, and unused land. Due to the severe salinization of farmland and grassland, and the large-scale disappearance of wetland in Western Jilin, saline-alkali land and marsh were separated from unused land in this study. Meanwhile, dryland and paddy field were separated from farmland. Through field verification, the interpretation precision was >94.3%, which could satisfy the accuracy requirement of 1:100,000 mapping. 

The topography elevation data, high-resolution raster DEM, were derived from the shuttle radar topography mission (SRTM) C-band data [26]. The DEM data with 90 m spatial resolution was processed into slope and aspect data.

The soil data came from the Institute of Soil Science, CAS, which provided the 1:1,000,000 scale Soil Map of China, including various soil attributes such as soil texture, organic carbon content, soil acidity, soil drainage ability and so on.

Meteorological data for 1975–2013, which included monthly mean minimum temperature, mean maximum temperature, cumulative precipitation, cumulative radiation, mean relative humidity, mean wind speed at 10 m height and wet day frequency. The data were obtained from 19 national meteorological stations maintained by the Chinese Meteorological Administration. The monthly data for the above seven key plant growth factors were interpolated to 1 km resolution by using ANUSPLIN software based on the DEM of Western Jilin [27,28,29]. Statistical data (such as actual soybean yields and irrigated percentage of each county) derived from Jilin Statistical Yearbook.

### 2.3. Methodology

#### 2.3.1. Crop Production Potential Simulation Method

Crop production potential was simulated using the GAEZ model. Over the past 30 years, the International Institute for Applied Systems Analysis (IIASA) and the Food and Agriculture Organization of the United Nations (FAO) have been continuously developing the AEZ methodology for assessing agricultural resources and potential [30]. Then, the GAEZ model has been developed. The GAEZ model estimated the climatic suitability of crops based on meteorological conditions and then calculated crop production potential step by step taking into account other factors, including light potential production (only limiting light), light and temperature potential production (limiting light and temperature), climatic potential production (limiting light, temperature, and water), land potential production (limiting light, temperature, water, soil and topography), and agricultural potential production (considering limiting agricultural input level and management measures) [31,32,33,34,35,36,37,38,39,40]. The detailed calculation procedures of GAEZ model includes six main steps of data processing, namely:

1. Module1: Climatic data analysis and compilation of general agro-climatic indicators. In Module1, seven climatic data of a specific year are input to the GAEZ model, and then the module calculates and stores climate-related variables and indicators for each grid-cell.
2. Module2: Grain-specific agro-climatic assessment and potential water-limited yield calculation. Module2 calculates the yield of all crop types with the specific climate conditions, considering the limit of water supply.
3. Module3: Yield-reduction due to agro-climatic constraints. This step is carried out to make explicit the effect of limitations due to soil workability, pest and diseases, and then revises the calculation results of Module2.
4. Module4: Edaphic assessment and yield reduction due to soil and topography limitations. This module evaluates yield reduction due to limitations imposed by soil and topography conditions.
5. Module5: Integration of results from Module1–4 into crop-specific grid-cell databases. This module reads the results of the agro-climatic evaluation for yield calculated in Module2/3 for different soil classes and it uses the edaphic rating produced for each soil/slope combination in Module4.
6. Module6: Actual yield and production. This module estimates actual yield and production of specific crop types according to the percentage of farmland area to each grid-cell area and shares of rain-fed and irrigated farmland within each grid-cell, using a downscaling method.

The detailed calculation process under Module1–6 of the GAEZ model has been given in Appendix A. Based on the potential cropping system calculated by the GAEZ model, crop production potential is determined considering various cropping systems (including double cropping per year, triple cropping for two years and triple cropping per year). However, Western Jilin has a long and cold winter, so the only cropping system in Western Jilin is single cropping per year.

In this study, soybean was planted in both irrigated and rain-fed scenarios. Calculations of the potential production for the rain-fed scenarios were based on light, temperature, and water conditions, whereas those for the irrigation scenarios only used light and temperature conditions, assuming sufficient water for crop growth and no water stress. The final crop potential was calculated according to the following formula within each grid-cell:

*production_t_* = *production_i_* × *i* + *production_r_* × (1 − *i*) (1)

where *production_t_* represents the total production potential within each grid-cell (kg/ha), *production_i_* represents the production potential under the scenarios in which all the farmland is irrigated land (kg/ha), *production_r_* is the production potential under rain-fed scenarios (kg/ha), and *i* indicates the percentage of irrigated land area to total farmland area [41]. 

**Figure 2:**
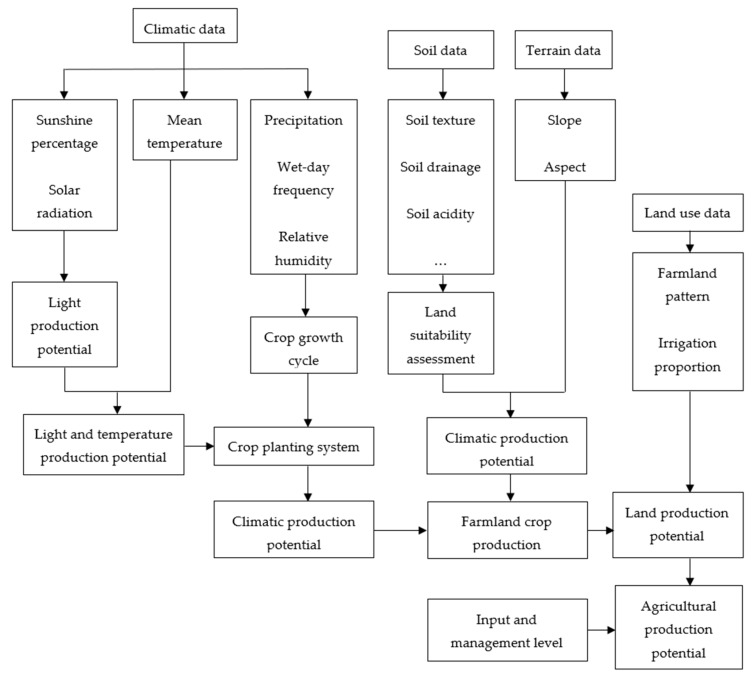
The flow chart of soybean production potential calculation using the Global Agro-Ecological Zones (GAEZ) model.

The flow chart of soybean production potential calculation using the GAEZ model in this study is shown in Figure 2.

#### 2.3.2. Farmland Change Impact Analysis on Soybean Production Potential 

Soybean production potential was greatly affected by farmland change. Farmland change included the conversion between dryland and other categories, and the change of irrigation percentage to dryland. The conversion between dryland and other categories included dryland expansion such as reclamation of grassland and forest, and dryland loss such as urban expansion and farmland salinization. In this study, we first analyzed the change of area and spatial distribution characteristics of dryland in Western Jilin between 1975–2000 and 2000–2013, and then considered the impact of farmland change on soybean production potential on the whole. Next, we assumed that, when the conversion between dryland and other categories or the irrigation percentage remained the same, how did the new condition affect soybean production potential.

## 3. Results and Analysis

### 3.1. Results Validation

**Figure 3:**
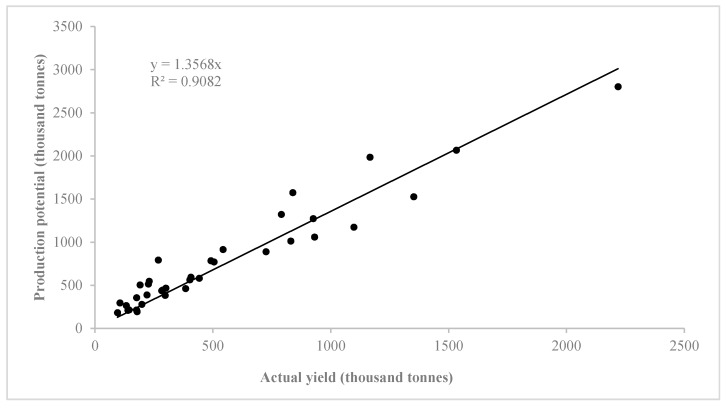
Comparison between production potential and actual yields in each county in 1975, 2000 and 2013.

To verify the accuracy of the simulation results of crop potential production in Western Jilin, we compared the production potential of major crops (including rice, maize and soybean, which account for more than 90% of total grain yields) of 12 counties in Western Jilin in 1975, 2000 and 2013 calculated by using the GAEZ model with actual statistical grain yields from Jilin Statistical Yearbook, setting up regression relation between potential production and actual statistical yields. The total crop production potential of the three years was 28.02 million tonnes, nearly 1.46 times the actual yields. The correlation between the calculated potential production and actual yield of each county is shown in Figure 3. The horizontal axis shows the actual yield of major crops in each county in three years, and the vertical axis shows the production potential of major crops in each county calculated by the GAEZ model. The cross-correlation coefficient was 0.82, indicating a good correlation. Consequently, the trend in calculated potential production reflected the trend in actual yields. This result could be used to explain the accuracy of the simulation results using the GAEZ model.

### 3.2. Spatial Contribution Characteristics of Soybean Production Potential in Western Jilin in 2013

Various factors could affect soybean production potential, and the climate and soil in Western Jilin have changed a lot from 1975 to 2013. However, in order to analyze impact of farmland change of Western Jilin from 1975 to 2013, we need to ensure that soil and topography factors were unchanged nearly 40 years, and used monthly mean meteorological data from 1975 to 2013 to analyze the change of soybean production potential led by farmland change. The soil data we used came from the 1:1,000,000 scale Soil Map of China. 

**Figure 4:**
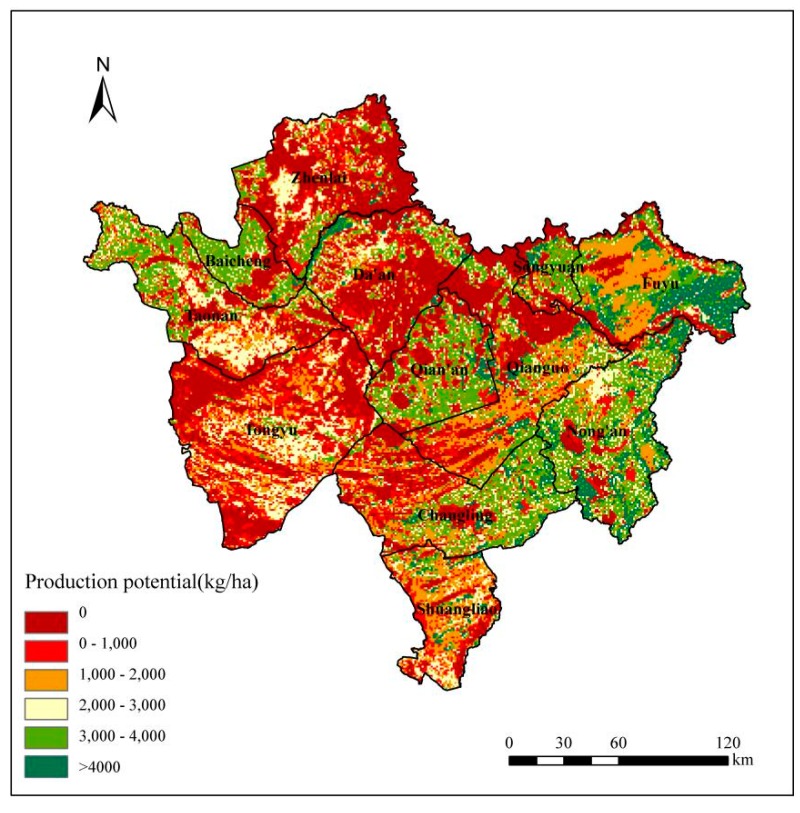
Spatial distribution of soybean production potential in 2013.

Using the GAEZ model, this study calculated soybean production potential in Western Jilin step by step taking into account meteorological data, soil data, DEM data and farmland data of 2013, and obtained the spatial distribution map of soybean production potential of 2013 (Figure 4). In 2013, the total soybean production potential of Western Jilin was 8.92 million tonnes, and the average soybean production potential was 1612 kg/ha (Figure 4). It can be seen from the spatial distribution of soybean production potential of Western Jilin in 2013 that the production potential of eastern area was higher than the other areas of Western Jilin. It was mainly because that the farmland density of the east was highest, and the topography, soil and climate conditions were more suitable for soybean production [42]. It may also be because that in west and south in Western Jilin, land salinization and desertification were serious, and the soil quality is poor [25], which was not conducive to the growth of soybean. 

**Figure 5:**
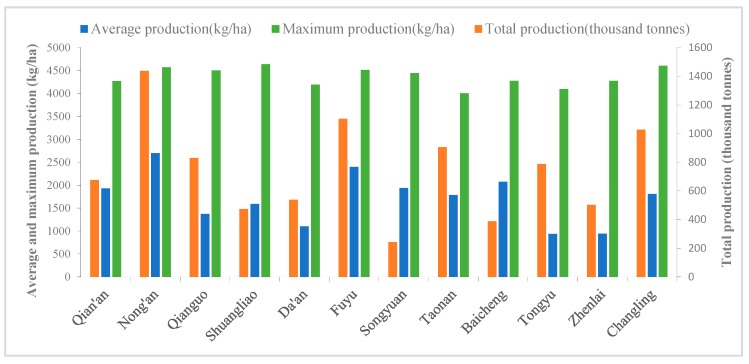
Soybean production potential in Western Jilin in 2013.

We used administrative areas data of Western Jilin and soybean production potential data calculated by the GAEZ model to count the average production, maximum production and total production of each county (Figure 5). Nong’an had the maximum total production potential of 1438 thousand tonnes and average production potential of 2701.68 kg/ha (Figure 5), where the soil was fertile and had suitable hydrothermal conditions and there were few land salinization and desertification. 

In contrast, the area that had the minimum total production potential was Songyuan at 243 thousand tonnes. It was mainly due to the small area of farmland. Tongyu had the minimum average production potential of 938.33 kg/ha, where there were low rainfall, low farmland density, and a large area of sand and saline-alkali land. The county with the maximum production potential was Shuangliao at 4639 kg/ha.

### 3.3. Impact of Farmland Change on Soybean Production Potential

#### 3.3.1. Characteristics of Farmland Change in Western Jilin between 1975–2013

**Figure 6:**
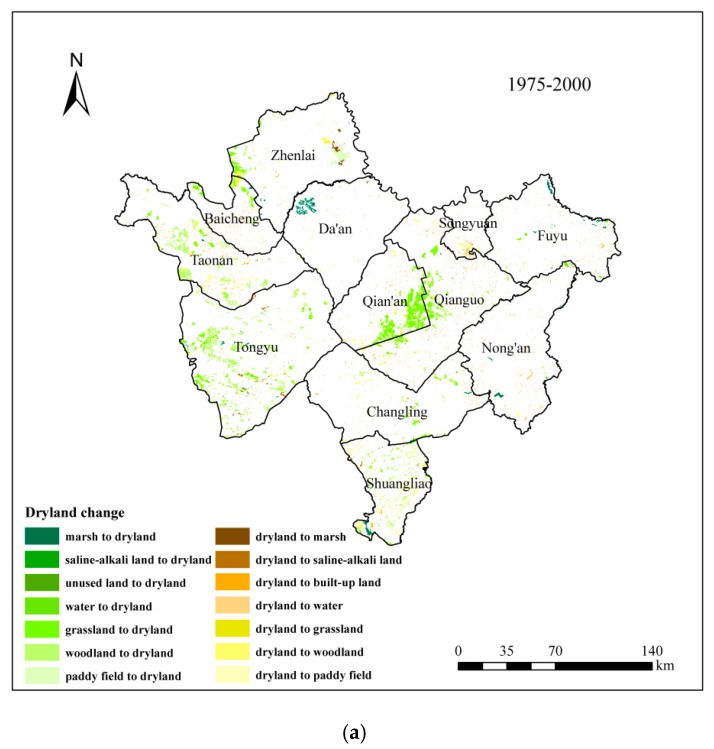

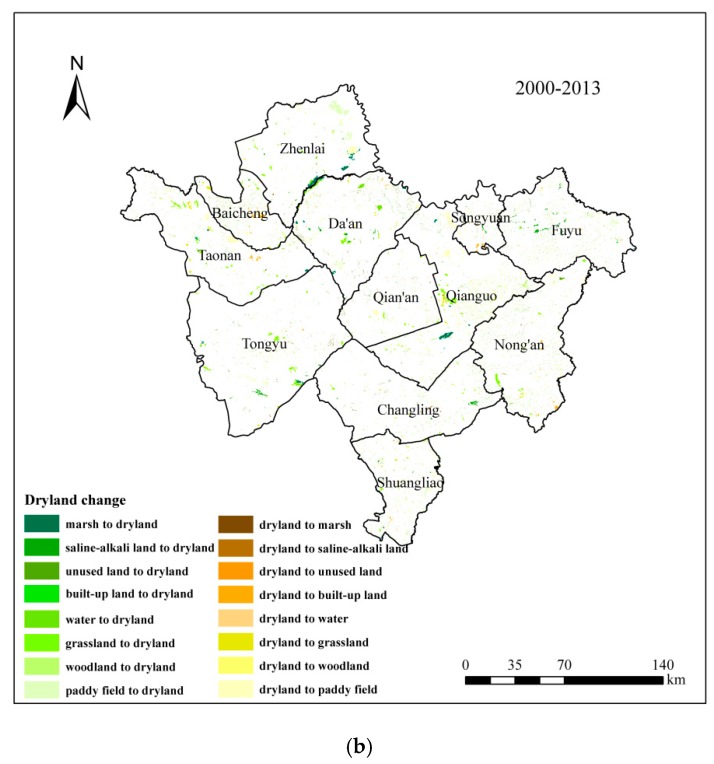
Farmland change between 1975 and 2000, and between 2000 and 2013 (**a**) between 1975 and 2000; (**b**) between 2000 and 2013.

We intersected the land use data of 1975 and 2000, and of 2000 and 2013 respectively by ArcGis, and then obtained the spatial distribution map of the conversion between dryland and other categories (Figure 6). The statistics are shown in Table 1 and Table 2. From 1975 to 2013, the total area of dryland in Western Jilin showed an increased trend from 1975 to 2000 (Figure 6a), and was nearly unchanged from 2000 to 2013 (Figure 6b).

During the first period, the area of dryland increased by 1360.9 km^2^, with an increase in the center and west of Western Jilin and a decrease in the north. The areas with an increasing dryland were mainly located in the east of Qian’an, the west of Zhenlai, and some areas of Taonan and Tongyu. The areas with a decreasing dryland were sporadically located in the whole area of Western Jilin. 

The increased dryland mainly came from grassland (1218.45 km^2^), woodland (717.72 km^2^), paddy field (159.58 km^2^) and marsh (154.37 km^2^). The decreasing dryland mainly converted into woodland (352.16 km^2^), built-up land (303.94 km^2^), and paddy field (104.67 km^2^) (Table 1). During this period, land reclamation was relatively intense, especially grassland and woodland converted to farmland, which accounted for 84.71% of the total increasing dryland. Meanwhile, built-up land expansion also led to part of the dryland reduction. The area of dryland increased by 224.70 km^2^ during the second period. There were 972.37 km^2^ of non-dryland converted into dryland and 747.67 km^2^ dryland converted into non-dryland.

The areas with a decreasing and increasing dryland were all sporadically located in the whole area of Western Jilin. The increasing dryland mainly came from grassland (261.35 km^2^), paddy field (157.49 km^2^), woodland (127.09 km^2^) and built-up land (124.35 km^2^). 

The decreasing dryland mainly converted into paddy field (310.46 km^2^), built-up land (168.75 km^2^) and woodland (126.26 km^2^) (Table 1). In this period, as Western Jilin began to consider environmental protection, there was widespread return of farmland to woodland and grassland, did not appreciably resulting in minimal overall dryland area increase.

#### 3.3.2. Impact of Farmland Change on Soybean Production Potential from 1975 to 2013

**Figure 7:**
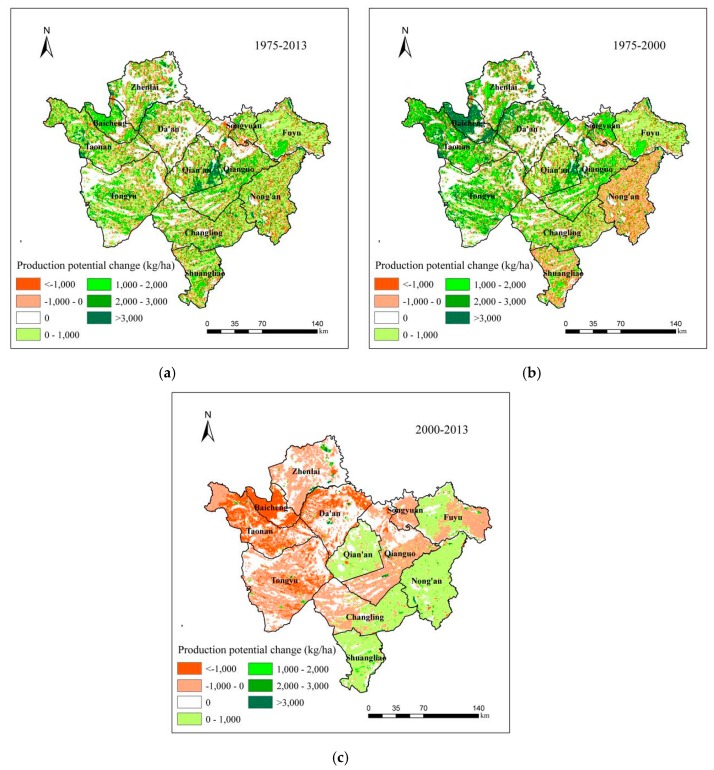
Soybean production potential change between 1975 and 2000, between 2000 and 2013, and between 1975 and 2013. (**a**) between 1975 and 2000; (**b**) between 2000 and 2013; (**c**) between 1975 and 2013.

In this study, using the GAEZ model, we calculated the soybean production potential in Western Jilinin in three years of 1975, 2000 and 2013, and then calculated the gap of soybean production potential during 1975–2013, and the first and second periods respectively (Figure 7). From 1975 to 2013, the characteristics of soybean production potential change led by farmland change are shown in Figure 7a. The average soybean production potential increased in most areas, especially in Baicheng (increased between 1000 and 2000 kg/ha) and the southeast of Qian’an (increased more than 3000 kg/ha) (Figure 7a). 

The net increase of soybean production potential was 22.73 thousand tonnes (Table 2 and Table 3). In general, the decrease of the soybean production potential was due to urban expansion and returning dryland to woodland and grassland, and the increase was mainly due to reclamation of woodland and grassland. The net increase of soybean production potential led by farmland change was 3.30 million tonnes during the first period (Figure 7b). The soybean production potential increased by 4.47 million tonnes and decreased by 1.17 million tonnes. The total increase in soybean production potential was about 3.8 times the total decrease. In the most areas of Western Jilin, such as Tongyu, Fuyu, Changling and Taonan, the average soybean production potential increased by less than 2000 kg/ha, and the average soybean production potential increased by more than 3000 kg/ha in Baicheng. However, in Nong’an and the most areas of Shuangliao, the average soybean production potential decreased by less than 1000 kg/ha (Figure 7b). In Table 2 from 1975 to 2000, the increase of soybean production potential was mainly due to reclamation of woodland and grassland, which occupied 96.09% of the total increase. Returning marsh to dryland was another important reason, especially in Shuangliao and Fuyu. The increase was mainly distributed in the center of Western Jilin, such as Qian’an, Qianguo and Changling. The decrease was mainly due to urban expansion and returning dryland to woodland and grassland, accounting for 31.59%, 30.14% and 22%, respectively, of the total decrease (Table 2). The soybean production potential in Qian’an increased by 1710 thousand tonnes, decreased by 48 thousand tonnes, and the net increase was 1662 thousand tonnes. The net increase in Qian’an was the first in Western Jilin, mainly due to the reclamation of woodland and grassland. The net increase of soybean production potential in Changling was 630 thousand tonnes, which was the second. In Zhenlai, the soybean production potential during the 25 years was nearly unchanged. In Songyuan, Taonan, and Fuyu, the soybean production potential decreased by 76, 42 and 21 thousand tonnes respectively (Table 2).

During the second period, the net decrease of soybean production potential led by farmland change was 1.03 million tonnes (Figure 7c). The soybean production potential increased by 0.68 million tonnes and decreased by 1.71 million tonnes. In the western and most central areas of Western Jilin, the average soybean production potential decreased by less than 1000 kg/ha, such as Zhenlai, Tongyu, Qianguo and Songyuan, and even the average soybean production potential decreased by more than 1000 kg/ha in Baicheng and Taonan. However, in Qian’an and the west of Fuyu, Nong’an, Shuangliao, and the east of Changling, the average soybean production potential increased by less than 1000 kg/ha. In Table 3, from 2000 to 2013, the decrease of soybean production potential was mainly due to urban expansion and returning dryland to woodland and grassland, which accounted for 85.59% of the total decrease, and it was also due to returning dryland to saline-alkali land, such as in Taonan and Tongyu. The increased production potential was still mainly distributed in the center of Western Jilin, such as Qian’an, and Qianguo, which were 99 thousand tonnes and 97 thousand tonnes respectively. The decreased production was distributed in all of the counties in Western Jilin, especially in Tongyu, Nong’an and Taonan, accounting for 12.01%, 10.92% and 10.49% respectively of the total decrease (Table 3). The net decrease of soybean production potential in Songyuan was 156 thousand tonnes, which was maximum. It was mainly due to the decrease of dryland caused by returning woodland and grassland. Qianguo was the only county where the soybean production potential increased during the second period (14 thousand tonnes) (Table 3). 

## 4. Discussions

### 4.1. Impact of Two Situations of Farmland Change on Soybean Production Potential

There are two situations that could cause farmland change, namely the conversion between dryland and other categories, and the change of irrigation percentage in dryland. In order to discuss the impact of two situations of farmland change on soybean production potential respectively, two scenarios were severally considered to calculate the soybean production potential of 2000 and 2013 once more. In Scenario I: the irrigation percentage in 2000 and 2013 was unchanged and the same as 1975, and only the land use categories changed between 1975 and 2013. In Scenario II: between 1975 and 2013, only the irrigation percentage changed, and the land use categories in 2000 and 2013 was unchanged and the same as 1975.

#### 4.1.1. Impact of the Conversion between Dryland and Other Categories 

**Figure 8:**
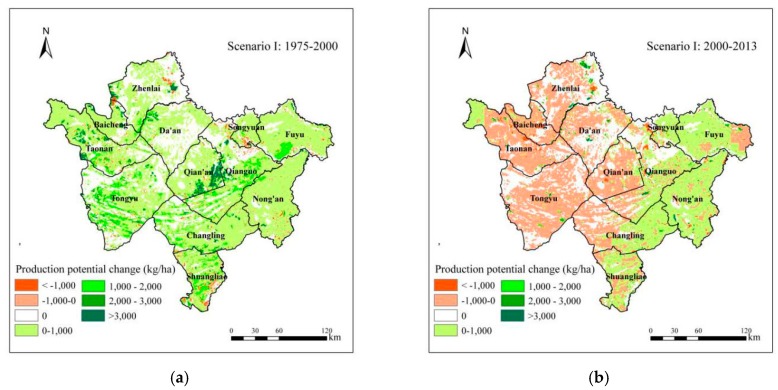
Spatial distribution of soybean production potential change between 1975 and 2000, and between 2000 and 2013 under Scenario I. (**a**) between 1975 and 2000; (**b**) between 2000 and 2013.

This study calculated soybean production potential in 1975, 2000, and 2013 using the GAEZ model under Scenario I (Figure 8). The total soybean production potential in 1975, 2000 and 2013 were 6.32, 8.63, and 8.75 million tonnes respectively. It indicated that the conversion between dryland and other categories led to the total soybean production potential in Western Jilin increased by 2.31 million tonnes between 1975 and 2000 (Figure 8a), and 0.12 thousand tonnes between 2000 and 2013 (Figure 8b). During 1975–2000, due to the sharp increase of dryland area, especially grassland and woodland reclamation, the average soybean production potential of the most areas in Western Jilin increased by less than 1000 kg/ha. But in the east of Qian’an, it increased by more than 2000 kg/ha due to grassland reclamation. In a few areas, the average soybean production potential decreased. During 2000–2013, it can be seen that in the west and center of Western Jilin, the average soybean production potential almost decreased by less than 1000 kg/ha, while in the east of Western Jilin, it almost increased by less than 1000 kg/ha. This conformed to the conversion characteristics of dryland between 2000 and 2013.

**Figure 9:**
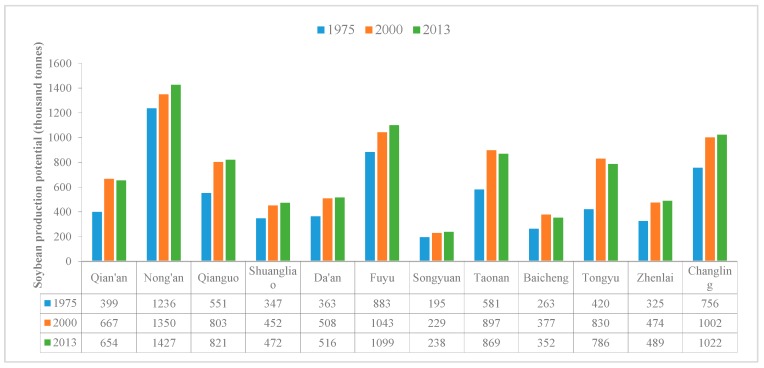
Soybean production potential in Western Jilin in 1975, 2000 and 2013 under Scenario I.

We counted soybean production potential of each county in Western Jilin in three years under Scenario I (Figure 9). In Western Jilin, compared with other counties, Nong’an had the maximum soybean production potential in 1975, 2000 and 2013, which were 1.24, 1.35 and 1.43 million tonnes respectively. Songyuan had the minimum production potential during this period, and it increased from 1975 (195 thousand tonnes) to 2013 (238 thousand tonnes). The production potential in Tongyu in 2000 (830 thousand tonnes) was about twice as many as in 1975 (420 thousand tonnes). Qian’an, Taonan, Baicheng and Tongyu were the four counties where the production potential decreased from 2000 to 2013 (Figure 9). 

#### 4.1.2. Impact of the Change of Irrigation Percentage

**Figure 10:**
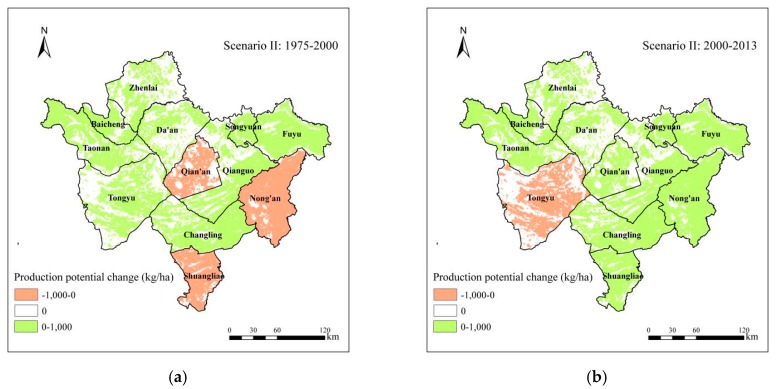
Spatial distribution of soybean production potential change between 1975 and 2000, and between 2000 and 2013 under Scenario II. (**a**) between 1975 and 2000; (**b**) between 2000 and 2013.

We also calculated soybean production potential in 1975, 2000, and 2013 using the GAEZ model under Scenario II (Figure 10). The total soybean production potential in 1975, 2000 and 2013 were 6.32, 6.60, and 6.89 million tonnes respectively, and the change range of average production potential was between −1000 and 1000 thousand tonnes. The change of irrigation percentage led to the total soybean production potential in Western Jilin increased by only 0.28 million tonnes between 1975 and 2000, and 0.29 million tonnes between 2000 and 2013. During 1975–2000, the average soybean production potential of the most areas in Western Jilin increased by less than 1000 kg/ha, but in Qian’an, Nong’an and Shuangliao, it decreased by less than 1000 kg/ha because of the decline in the irrigation percentage (Figure 10a). During 2000–2013, it can be seen that in Tongyu, the average soybean production potential decreased by less than 1000 kg/ha, while in the other counties of Western Jilin, it increased by less than 1000 kg/ha (Figure 10b).

**Figure 11:**
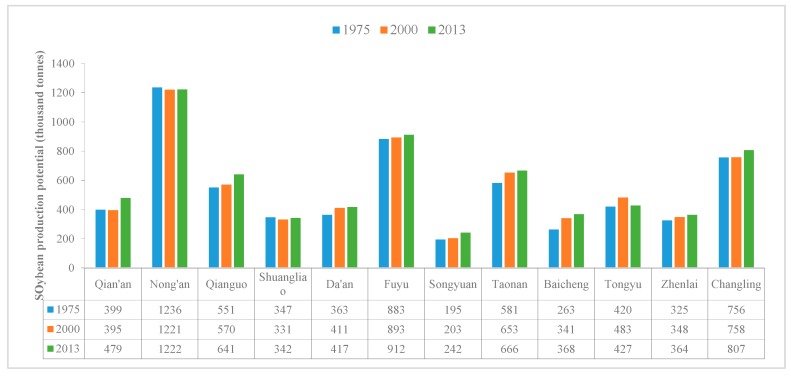
Soybean production potential in Western Jilin in 1975, 2000 and 2013 under Scenario II.

We also counted soybean production potential of each county in Western Jilin in three years under Scenario II (Figure 11). The soybean production potential of each county in Western Jilin was almost unchanged during 1975–2013 under Scenario II. Nong’an still had the maximum soybean production potential (1236 thousand tonnes in 1975, 1221 thousand tonnes in 2000, and 1222 thousand tonnes in 2013). Songyuan had the minimum production potential during 1975–2013 compared with other counties, which only increased from 1975 (195 thousand tonnes) to 2013 (242 thousand tonnes) (Figure 11). 

### 4.2. Comparison between the GAEZ Model and Other Methods

There are also many other crop production methods to calculate crop production potential. We take the WOFOST model and EPIC model as examples. First, the WOFOST simulation model is one method for analyzing the growth and production of crops under a wide range of weather and soil conditions. Such an analysis is important first to assess to what extent crop production is limited by the factors of light, moisture and macro-nutrients, and second to estimate what improvements are possible [43,44]. 

Second, the EPIC plant growth model is another method that was developed to estimate soil productivity as affected by erosion throughout the U.S. Since soil productivity is expressed in terms of crop yield, the model must be capable of simulating crop yields realistically for soils with a wide range of erosion damage [45]. The model can accurately describe the effects of light, temperature and water on crop growth and development. 

However, besides light, temperature, water and soil, topography is also an important fact to determine crop growth. For instance, steep irregular slopes are not practical for cultivation. Also, compared with other slope aspects, the south slope is more conducive to crop growth due to the large amount of solar radiation received. In the WOFOST model and EPIC model, topography is not considered to calculate crop production potential. The GAEZ model takes light, temperature, water, soil and topography into account. Compared with other methods, we can obtain more accurate results by using the GAEZ model. Therefore, we chose the GAEZ model to calculate soybean production potential in this study. 

### 4.3. Limitations of the GAEZ Model

In this study, we found that the GAEZ model also has some limitations. First, in this study the meteorological data for 1975–2013 were obtained from 19 national meteorological stations maintained by the Chinese Meteorological Administration and were interpolated to produce a continuous surface. However, it was difficult to obtain a very high resolution and accurate spatial distribution map of temperature, precipitation, wind speed, and so on by spatial interporation because the meteorological stations were scarce within the region [46]. Integration of meteorological data to improve the accuracy of spatial interpolation needs further consideration in the future. Besides, monthly mean climatic data were integrated into the GAEZ model, but extreme climate conditions such as extreme temperature and precipitation, may have had large effects on crop potential production. For example, by referring to the *Meteorological Disaster Yearbook of Liaoning* of 2013, severe drought ocurred in Western Jilin in the summer. Due to the drought, the total grain yield of 2013 was significantly lower than the total grain yields of 2012 and 2014 by referring to the *Liaoning Statistical Yearbooks* of 2012, 2013 and 2014. This indicated that severe drought could lead to reduction of crop yields. However, in this study we used monthly mean meteorological data from 1975 to 2013, so the apparent impact of the flooding on soybean production potential could not be considered.

Second, although it was assumed that the water supply was sufficient for crop growth under irrigation scenario in the GAEZ model, the actual availability of water provided by irrigation may be still limited for crop growth in actual practice [1]. For example, in this study, it was assumed that the water needed for soybean growth under irrigation conditions was sufficient. In fact, although some irrigation measures, such as sprinkler irrigation and drip irrigation, etc., could greatly ensure the water supply of crop growth, they could not ensure that all of the moisture for crop growth could be provided. This would lead to higher soybean production potential calculated by the GAEZ model compared with the actual yield.

Third, levels of input and management are also crucial factors for production potential, such as the use of optimum applications of nutrients and chemical pest, disease and weed control, as well as mechanized production. In fact, levels of input and management could not be always ideal. Due to the difficulty of quantitative research to input and management, it was assumed to be an optimal condition in the GAEZ model, which could also lead to higher soybean production potential compared with the actual yield. 

Fourth, in this study, in order to study the impact of farmland change on soybean production potential, other factors need to be unchanged, so the soil data used in this study came from the 1:1,000,000 scale Soil Map of China made in 2009 and was the same in recent 40 years. However, Soil characteristics, such as soil texture, soil acidity, soil organic carbon content, soil salinity would change over time that could have significant impact on crop production potential. Therefore, future work should focus more on soil change to study crop production potential. Therefore, how can this model be better improved so that it can be more accurate to calculate crop production potential, still need to be studied.

### 4.4. Advantage of Impact of Farmland Change on Production Potential Analysis Method

In general, changes of various factors could affect crop production potential, such as farmland, climate, soil, topography, and levels of input and management. However, in order to study the effect of farmland change separately, we have to ensure that other variables are unchanged. Besides, due to the two situations of farmland change, we have to make sure that one situation is unchanged in order to analyze the effect of the other situation change on soybean production potential. The method is called “Control Variate Method”. The advantage of this method is to ensure that the results are not disturbed by other factors. Therefore, this study can directly show the impact of farmland change on soybean production potential and the results were accurate relatively.

## 5. Conclusions

This study intended to analyze the impact of farmland change on soybean production potential in Western Jilin from 1975 to 2013. We first analyzed the change of dryland quality and spatial distribution during 1975–2013 in Western Jilin, and then used the GAEZ model to study its impact on soybean production potential. 

From 1975 to 2000, the total area of dryland in Western Jilin increased by 1360.91 km^2^ and increased by 224.70 km^2^ from 2000 to 2013. Farmland change led to a net increase of 3.30 million tonnes in soybean production potential between 1975 and 2000, and a net decrease of 1.03 million tonnes between 2000 and 2013. During the first period, the increase of soybean production potential was mainly due to reclamation of woodland and grassland, which occupied 96.09% of the total increase. During the second period, the decrease of soybean production potential was mainly because of urban expansion and returning dryland to woodland and grassland, which accounted for 85.59 % of the total decrease. 

We also considered two situations that caused farmland change respectively, namely the conversion between dryland and other categories, and the change of irrigation percentage. The conversion between dryland and other categories led to the total soybean production potential in Western Jilin increased by 2.31 million tonnes between 1975 and 2000, and 0.12 million tonnes between 2000 and 2013. The irrigation percentage was also an important aspect that could affect the production potential, which led to the total soybean production potential in Western Jilin increased by only 0.28 million tonnes during the first period, and 0.29 million tonnes between 2000 and 2013.

Overall, the area of farmland in Western Jilin increased rapidly before 2000. After 2000, Western Jilin paid more attention to ecological environment preservation, and some farmland was changed to non-agricultural utilization mode, resulting in the slow growth of farmland area. Therefore, optimizing the structure and distribution of land use, improving quality of farmland, and correctly analyzing soybean production potential and its regional differences and impact of farmland change on soybean production potential, are good measures to raising the conversion rate of soybean potential production to actual yield. It’s also of great significance to protect safe baseline of farmland, manage land resources, and ensure continuity and stability of soybean supply and food security. 

## Figures and Tables

**Table 1:** The area of farmland change between 1975 and 2000, and between 2000 and 2013 (km^2^).

Dryland Change	Dryland Change Types	1975–2000	2000–2013
Dryland decrease	Dryland to paddy field	104.67	310.46
	Dryland to woodland	352.16	126.26
	Dryland to grassland	77.75	81.10
	Dryland to water	12.54	9.94
	Dryland to built-up land	303.94	168.75
	Dryland to saline-alkali land	43.39	46.37
	Dryland to marsh	29.91	3.27
	Dryland to unused land	0.00	1.52
	Total decrease	924.36	747.67
Dryland increase	Paddy field to dryland	159.58	157.49
	Woodland to dryland	717.72	127.09
	Grassland to dryland	1218.45	261.35
	Water to dryland	15.43	80.69
	Built-up land to dryland	0.00	124.35
	Unused land to dryland	9.63	5.20
	Saline-alkali land to dryland	10.09	112.66
	Marsh to dryland	154.37	103.54
	Total increase	2285.27	972.37
Total change		1360.91	224.70

**Table 2:** Impact of farmland change on soybean production potential in Western Jilin during 1975–2000 (thousand tonnes).

Dryland Change	Dryland Change Types	Qian’an	Nong’an	Qianguo	Shuangliao	Da’an	Fuyu	Songyuan	Taonan	Baicheng	Tongyu	Zhenlai	Changling	Total
Decrease	Returning dryland to paddy field	−11	0	0	−27	−11	−31	−21	0	0	0	0	0	−101
	Returning forests	0	0	−22	−21	−45	−21	−33	−71	−46	−37	−34	−22	−352
	Returning grassland	−26	0	−25	−33	0	0	0	−13	−47	−71	−42	0.00	−257
	Returning water	0	0	0	0	0	0	0	0	0	0	0	0	0
	Urban expansion	−11	−69	−29	−34	−28	−13	−22	−40	−36	−45	0	−42	−369
	Returning dryland to saline-alkali land	0	−26	−2	0	0	0	0	−22	0	−12	0	0	−62
	Returning dryland to marsh	0	0	0	0	0	0	0	0	0	−13	−13	0.00	−26
	Total decrease	−48	−95	−78	−115	−84	−65	−76	−147	−129	−178	−89	−64	−1168
Increase	Returning paddy field to dryland	0	0	3	0	0	0	0	9	9	0	25	0	46
	Woodland reclamation	797	179	58	14	57	6	0	78	49	52	11	16	1317
	Grassland reclamation	913	59	446	98	123	12	0	13	241	337	60	677	2979
	Returning water to dryland	0	2	0	0	0	1	0	0	0	0	0	0	3
	Unused land reclamation	0	0	0	0	0	0	0	0	0	1	0	0	1
	Returning saline-alkali land to dryland	0	0	2	8	0	2	0	0	0	2	0	0	14
	Returning marsh to dryland	0	16	18	25	13	23	0	5	1	7	1	1	110
	Total increase	1710	257	527	145	193	44	0	105	300	399	97	694	4471
Net change		1662	162	449	30	109	−21	−76	−42	171	221	8	630	3303

**Table 3:** Impact of farmland change on soybean production potential in Western Jilin during 2000–2013 (thousand tonnes).

Dryland Change	Dryland Change Types	Qian’an	Nong’an	Qianguo	Shuangliao	Da’an	Fuyu	Songyuan	Taonan	Baicheng	Tongyu	Zhenlai	Changling	Total
Decrease	Returning dryland to paddy field	0	−13	−11	−24	−21	0	−43	0	0	−21	0	0	−133
	Returning forests	−31	−32	0	−36	−54	−47	−35	−69	−56	−43	−39	−47	−489
	Returning grassland	−56	−41	−31	−34	−12	−21	−31	−39	−27	−74	−45	−43	−454
	Returning water	0	0	0	0	0	0	0	0	0	0	0	0	0
	Urban expansion	−32	−74	−41	−45	−31	−55	−47	−42	−45	−36	−41	−29	−518
	Returning dryland to saline-alkali land	−1	−23	0	0	−2	0	0	−29	0	−28	0	0	−83
	Returning dryland to marsh	0	0	0	0	0	−11	0	0	0	−3	−16	0	−30
	Total decrease	−120	−183	−83	−139	−120	−134	−156	−179	−128	−205	−141	−119	−1707
Increase	Returning paddy field to dryland	0	0	2	0	0	0	0	5	4	0	17	0	28
	Woodland reclamation	48	24	13	15	24	0	0	51	24	25	7	30	231
	Grassland reclamation	51	21	74	51	32	0	0	2	27	32	45	32	367
	Returning water to dryland	0	0	0	0	0	0	0	0	0	0	0	0	0
	Unused land reclamation	0	0	0	0	0	0	0	0	1	0	0	0	1
	Returning saline-alkali land to dryland	0	0	0	2	0	2	0	0	0	2	0	0	6
	Returning marsh to dryland	0	0	8	15	3	13	0	2	0	2	0	1	44
	Total increase	99	45	97	83	59	15	0	60	56	61	69	63	707
Net change		−21	−138	14	−56	−61	−119	−156	−119	−72	−144	−72	−56	−1000

## Appendix A

The detail calculation equations of the GAEZ model are the following.

**1. Module 1: Climatic Data Analysis and Compilation of General Agro-Climatic Indicators**

In Module 1, climatic variables are prepared for the use in GAEZ through conversions and temporal interpolations. The gridded monthly climatic variables was interpolated into daily data, providing the basis for the calculation of soil water balances and agro-climatic indicators relevant to plant production.

**(1) Wet Day Frequency**

Wet day frequency (WET) is used to derive daily precipitation events from monthly totals:

(A1) $$\;WET=WET^{ref}\;\times \;{\left(\frac{P}{P^{ref}}\right)}^{0.45}\;$$

where *P* and *P^ref^* are respectively the monthly precipitation of the historical or future time periods and monthly precipitation of the 1961–1990 reference climate period. *WET^ref^* represents the monthly wet day frequency in the reference climate.

**(2) Sunshine Duration**

Actual sunshine duration (n) is used for the calculation of incoming solar radiation, for evapotranspiration, and biomass calculations. Sunshine duration is calculated from the ratio actual sunshine hours over maximum possible sunshine hours (n/N).

**(3) Day-Time and Night-Time Temperatures**

The temperature during day-time (*Tday*, °C) and night-time (*Tnight*, °C) are calculated as follows:

(A2) $$\;Tday=Ta+\left(\frac{Tx-Tn}{4\pi }\right)\times \left(\frac{11+T_{0}}{12-T_{0}}\right)\times \sin\left(\pi \times \left(\frac{11-T_{0}}{11+T_{0}}\right)\right)\;$$

Night-time temperature is calculated as:

(A3) $$\;Tnight=Ta-\left(\frac{Tx-Tn}{4\pi }\right)\times \left(\frac{11+T_{0}}{T_{0}}\right)\times \sin\left(\pi \times \left(\frac{11-T_{0}}{11+T_{0}}\right)\right)\;$$

where *Ta* is average 24 h temperature, and *T*_0_ is calculated as a function of day-length (*DL*, hours):

(A4) $$\;T_{0}=12-0.5\times DL\;$$

Day-length is calculated in the model and depends on the latitude of a grid-cell and the day of the year.

**(4) Reference Evapotranspiration (ETo)**

The reference evapotranspiration (ETo) represents evapotranspiration from a defined reference surface, which closely resembles an extensive surface of green, well-watered grass of uniform height (12 cm), actively growing and completely shading the ground. The calculation procedure uses a standardized set of input parameters, as follows:

*T_max_* maximum daily temperature (°C)
*T_min_* minimum daily temperature (°C)
*RH* mean daily relative humidity (%)
*U2* wind speed measurement (ms^−1^)
*SD* bright sunshine hours per day (hours)
*A* elevation (m)
*L* latitude (deg)
*J* Julian date, i.e., number of day in year

The *Penman-Monteith combination equation* can be written in terms of an aerodynamic and a radiation term:

(A5) $$\;ET_{o}=ET_{ar}+ET_{ra}\;$$

where the *aerodynamic term* can be approximated by

(A6) $$\;ET_{ar}=\frac{\gamma }{\vartheta +\gamma^{*}}\times \frac{900}{T_{a}+273}\times U2\times \left(e_{a}-e_{d}\right)\;$$

and the radiation term by

(A7) $$ET_{ra}=\frac{\vartheta }{\vartheta +\gamma^{*}}\times \left(R_{n}-G\right)\times \frac{1}{\lambda }.\;$$

where variables in (A6) and (A7) are as follows:

*T_a_* average daily temperature (°C)
*e_a_* saturation vapor pressure (kPa)
*e_d_* vapor pressure at dew point (kPa)
$\left(e_{a}-e_{d}\right)$ vapor pressure deficit (kPa)
*R_n_* net radiation flux at surface (MJ m^−2^ d^−1^)
*G* soil heat flux (MJ m^−2^ d^−1^)

In the calculation procedure for the reference crop we use the following relationships to define terms in (A6):

*Arage daily temperature:*

(A8) $$\;T_{a}=0.5\left(T_{\max}+T_{\min}\right)\;$$

*Latent heat of vaporization*:

*λ* = 2.501 − 0.002361*T_a_* (A9)

*Atmospheric pressure (kPa) at elevation A:*

(A10) $$\;P=101.3{\left(\frac{293-0.0065A}{293}\right)}^{5.256}\;$$

*Psychrometric constant:*

(A11) $$\;r=0.0016286\cdot \frac{P}{\lambda }$$

*Aerodynamic resistance:*

(A12) $$\;r_{a}=\frac{208}{U2}\;$$

*Crop canopy resistance:*

(A13) $$r_{c}=\frac{R_{l}}{0.5LAI}$$

where under ambient CO_2_ concentrations the average daily stomata resistance of a single leaf, *R_l_* (sm^−1^), is set to *R_l_* = 100, and leaf area index of the reference crop is assumed as *LAI* = 24 ⋅ 0.12 = 2.88.

*Modified psychrometric constant:*

(A14) $$\gamma^{*}=\left(1+\frac{r_{c}}{r_{a}}\right)$$

*Saturation vapor pressure e_a_* for given temperatures *T*_min_ and *T*_max_

(A15) $$e_{ax}=0.6108\exp\left(\frac{17.27\;T_{\max}}{237.3+T_{\max}}\right)$$

(A16) $$e_{an}=0.6108\exp\left(\frac{17.27\;T_{\min}}{237.3+T_{\min}}\right)$$

(A17) $$\;e_{a}=0.5\;\left(e_{ax}+e_{an}\right)\;$$

*Vapor pressure* at dew point,$\;e_{d}$:

(A18) $$e_{d}=\frac{RH}{100}\cdot \frac{0.5}{\left(\frac{1}{e_{ax}}+\frac{1}{e_{an}}\right)}$$

*Slope of vapor pressure curve*, for given temperatures *T*_max_ and *T*_min_:

(A19) $$\vartheta_{x}=\frac{4096\;e_{ax}}{{\left(237.3+T_{\max}\right)}^{2}}$$

(A20) $$\vartheta_{n}=\frac{4096\;e_{an}}{{\left(237.3+T_{\min}\right)}^{2}}$$

(A21) $$\vartheta =\left(\vartheta_{x}+\vartheta_{n}\right)$$

Using (A8)–(A21) all variables in (A6) can be calculated from the input parameters. To determine the remaining variables $R_{n}$ and G used in the radiation term $ET_{ra}$ of Equation (A7), we proceed with the following calculation steps:

*Latitude* expressed in rad:

(A22) $$\;\varphi =\frac{L\pi }{180}\;$$

*Solar declination* (rad):

(A23) $$\;\delta =0.4093\sin\left(\frac{2\pi }{365}J-1.405\right)\;$$

*Relative distance* Earth to Sun:

(A24) $$d=1+0.033\cos\left(\frac{2\pi }{365}J\right)$$

*Sunset hour angle* (rad):

(A25) ψ=arccos(−tanφtanδ) 

*Extraterrestrial radiation* (MJ m^−2^d^−1^):

(A26) $$\;R_{a}=37.586\;d\;(\psi \sin\varphi \sin\delta +\;\cos\varphi \cos\delta \sin\psi )\;$$

*Maximum daylight hours:*

(A27) $$DL=\frac{24}{\pi }\psi$$

*Short-wave radiation*$R_{s}$ (MJ m^−2^d^−1^)

(A28) $$R_{s}=\left(0.25+0.5\frac{SD}{DL}\right)R_{a}$$

For a reference crop with an assumed albedo coefficient *α* = 0.23 *net incoming short-wave radiation* $R_{ns}$ (MJ m^−2^d^−1^) is:

(A29) $$R_{ns}=0.77\;R_{s}$$

*Net outgoing long-wave radiation*$R_{nl}$ (MJ m^−2^d^−1^) is estimated using:

(A30) $$R_{nl}=4.903\cdot 10^{-9}\left(0.1+0.9\frac{SD}{DL}\right)(0.34-0.139\sqrt{e_{d}})\frac{{\left(273.16+T_{\max}\right)}^{4}+{\left(273.16+T_{\min}\right)}^{4}}{2}\;$$

Using (A25) and (A26), *net radiation flux* at surface, $R_{n}$, becomes

*R_n_* = *R_ns_* − *R_nl_* (A31)

Finally, *soil heat flux* is approximated using

(A32) $$G=0.14\left(T_{a,n}-T_{a,n-1}\right)$$

where $T_{a,n}$ and $T_{a,n-1}$ are average monthly temperatures of current and previous month, respectively. With Equations (A9), (A14), (A21), (A31) and (A32) all variables in (A7) are defined and can be calculated from the input parameters described at the beginning of this Appendix.

**(5) Maximum Evapotranspiration (ETm)**

In Module I, the calculation of evapotranspiration (*ETm*) for a ‘reference crop’ assumes that sufficient water is available for uptake in the rooting zone. The value of *ETm* is related to *ETo* through applying crop coefficients for water requirement (*kc*). The *kc* factors are related to phenological development and leaf area. The *kc* values are crop and climate specific. They vary generally between 0.4–0.5 at initial crop stages (emergence) to 1.0–1.2 at reproductive stages:

(A33) ETm=kc×ETo

**(6) Actual Evapotransiration (ETa)**

The actual uptake of water for the ‘reference’ crop is characterized by the actual evapotranspiration (*ETa*, mm/day). The calculation of *ETa* differentiates two possible cases depending on the availability of water for plant extraction:

(i) Adequate soil water availability (*Eta* = *ETm*)
(ii) Limiting soil water availability (*Eta* < *ETm*)

When water is not limiting, the *ETa* value is equal to the maximum evapotranspiration (*ETm*) of the ‘rewference’ crop. At limiting water conditions, *ETa* is a fraction of *ETm*, depending on soil water availability as explained in following sections.

**(7) Snow Balance Calculation**

The calculation of a snow balance (*Sb*, mm) affects the water balance procedure outlined above. The snow balance increases when precipitation falls as snow and decreases with snowmelt and snow sublimation. All precipitation (*P*) falls as snow (*P^snow^*) when maximum temperature (*Tx*) is below a temperature threshold (*Ts*).

Snowmelt (*Sm*) is calculated as a function of daily maximum temperature, the snow melt parameter (*δ*) and is subject to the previously accumulated snow balance. The snow melt factor δ is set to 5.5 mm/°C.

(A34) Sm=min(δ×(Tx−Ts), Sb) 

The sublimation factor (*ks*) is used to discount a fraction of maximum evapotranspiration as sublimated snow. This fraction (*ks* × *ETm*) is subtracted from the snow balance:

(A35) $$\;Sb_{j}=Sb_{j-1}-Sm-\left(ks\times ETm\right)+P^{snow}\;$$

The sublimation factor (*ks*) is assumed to be 0, 0.1 or 0.2 of reference evapotranspiration (*ETm*, mm), depending temperature:

*ks = 0.0* *when Tx < Ts; Ts is assumed as 0 °C in GAEZ*
*ks = 0.1* *when Tx > Ts and Ta < 0 °C*
*ks = 0.2* *when Tx > Ts and 0 °C < Ta < 5 °C*

**2. Module 2: Grain-Specific Agro-Climatic Assessment and Potential Water-Limited Yield Calculation**

In this section the calculation procedures of constraint-free biomass and yield (i.e., carbon accumulation driven mainly by prevailing radiation and temperature regimes in a grid-cell) are explained. The constraint-free crop yields calculated in the AEZ biomass model reflect yield potentials with regard to temperature and radiation regimes prevailing in the respective grid-cells.

The AEZ methodology for the calculation of potential net biomass and yields is based on eco-physiological principles, as outlined below:

To calculate the net biomass production (*B_n_*) of a crop, an estimation of the gross biomass production (*B_g_*) and respiration loss (*R*) is required:

*B_n_* = *B_g_* − *R* (A36)

The equation relating the rate of net biomass production (*b_n_*) to the rate of gross biomass production (*b_g_*) and the respiration rate (*r*) is:

*b_n_* = *b_g_* − *r* (A37)

The maximum rate of net biomass production (*b_nm_*) is reached when the crop fully covers the ground surface. The period of maximum net crop growth, i.e., the point in time when maximum net biomass increments occur, is indicated by the inflection point of the cumulative growth curve. When the first derivative of net biomass growth is plotted against time the resulting graph resembles a normal distribution curve. The model assumes that the average rate of net production (*b_na_*) over the entire growth cycle is half the maximum growth rate, i.e., *b_na_* = 0.5 *b_nm_*. The net biomass production for a crop of N days (*B_n_*) is then:

*B_n_* = *0.5 b_nm_* × *N* (A38)

The maximum rate of gross biomass production (*b_gm_*) is related to the maximum net rate of CO_2_ exchange of leaves (*P_m_*) which is dependent on temperature, the photosynthesis pathway of the crop, and the level of atmospheric CO_2_ concentration.

For a standard crop, i.e., a crop in adaptability group I with *P_m_* = 20 kg ha^−1^ hr^−1^and a leaf area index of LAI = 5, the rate of gross biomass production *b_gm_* is calculated from the equation:

*b_gm_* = *F* × *b_o_* + (1 − *F*) *b_c_* (A39)

where:

F = the fraction of the daytime the sky is clouded, *F* = (*A_c_* − 0.5 *R_g_*)/(0.8 *A_c_*), where *A_c_* (or PAR) is the maximum active incoming short-wave radiation on clear days (de Wit, 1965), and *R_g_* is incoming short-wave radiation (both are measured in cal cm^−2^ day^−1^)
bo = gross dry mater production rate of a standard crop for a given location and time of the year on a completely overcast day, (kg ha^−1^ day^−1^) (de Wit, 1965)
bc = gross dry mater production rate of a standard crop for a given location and time of the year on a perfectly clear day, (kg ha^−1^ day^−1^) (de Wit, 1965)

When *P_m_* is greater than 20 kg ha^−1^ hr^−1^, *b_gm_* is given by the equation:

*b_gm_* = *F* (0.8 + 0.01*P_m_*) *b_o_* + (1 − *F*) (0.5 + 0.025 *P_m_*) *b_c_* (A40)

When *P_m_* is less than 20 kg ha^−1^ hr^−1^, *b_gm_* is calculated according to:

*b_gm_* = *F* (0.5 + 0.025 *P_m_*) *b_o_* + (1 − *F*) (0.05 *P_m_*) *b_c_* (A41)

To calculate the maximum rate of net biomass production (*b_nm_*), the maximum rate of gross biomass production (*b_gm_*) and the rate of respiration (*r_m_*) are required. Here, growth respiration is considered a linear function of the rate of gross biomass production [47], and maintenance respiration a linear function of net biomass that has already been accumulated (*B_m_*) When the rate of gross biomass production is *b_gm_*, the respiration rate *r_m_* is:

*r_m_* = *k b_gm_* + *c B_m_* (A42)

where *k* and *c* are the proportionality constants for growth respiration and maintenance respiration respectively, and *B_m_* is the net biomass accumulated at the time of maximum rate of net biomass production. For both legume and non legume crops *k* equals 0.28. However, *c* is temperature dependent and differs for the two crop groups. At 30 °C, factor *c*30 for a legume crop equals 0.0283 and for a non-legume crop 0.0108. The temperature dependence of *c_t_* for both crop groups is modelled with a quadratic function:

*c_t_ = c*30 (0.0044 *+* 0.0019 *T +* 0.0010 *T*^2^)*.* (A43)

It is assumed that the cumulative net biomass *B_m_* of the crop (i.e., biomass at the inflection point of the cumulative growth curve) equals half the net biomass that would be accumulated at the end of the crop’s growth cycle. Therefore, we set *B_m_* = 0.5 *B_n_*, and using (3), *B_m_* for a crop of *N* days is determined according to:

*B_m_* = 0.25 *b_nm_* × *N* (A44)

By combining the respiration equation with the equation for the rate of gross photosynthesis, the maximum rate of net biomass production (*b_nm_*) or the rate of net dry matter production at full cover for a crop of *N* days becomes:

*b_nm_* = 0.72 *b_gm_*/(1 + 0.25 *c_t_ N*) (A45)

Finally, the net biomass production (*B_n_*) for a crop of *N* days, where 0.5 *b_nm_* is the seasonal average rate of net biomass production, can be derived as:

*B_n_* = (0.36 *b_gm_* × L)/(1/*N* + 0.25 *c_t_*) (A46)

where:

*b_gm_* = maximum rate of gross biomass production at leaf area index (LAI) of 5
*L* = growth ratio, equal to the ratio of *b_gm_* at actual LAI to *b_gm_* at LAI of 5
*N* = length of normal growth cycle
*c_t_* = maintenance respiration, dependent on both crop and temperature according to Equation (A8)

Potential yield (*Y_p_*) is estimated from net biomass (*B_n_*) using the equation:

*Y_p_* = *H_i_* × *B_n_* (A47)

where:

*H_i_* = harvest index, i.e., proportion of the net biomass of a crop that is economically useful

Thus, climate and crop characteristics that apply in the computation of net biomass and yield are: (a) heat and radiation regime over the crop cycle, (b) crop adaptability group to determine applicable rate of photosynthesis *P_m_*, (c) length of growth cycle (from emergence to physiological maturity), (d) length of yield formation period, (e) leaf area index at maximum growth rate, and (f) harvest index.

**3. Module 3: Yield-Reduction due to Agro-Climatic Constraints**

The values of the yield reducing factors for agro-climatic constraints are systematically organized in look up tables accessed by GAEZ accordingly to:

(1) Land utilization type, LUT
(2) Thermal climate
(3) Input level
(4) Length of the growing period, LGP, length of the equivalent LGP (LGP_eq_), and the frost-free period (LGP_t=10_)

By combining the five agro-climatic yield reducing factors $fct_{a},\ldots ,fct_{e}$ for constraint types ‘a’ to ‘e’, an overall yield reducing factor (*fc*_3_) is calculated:

(A48) $$\;fc_{3}=\min\left\{\left(1-fct_{a}\right)\times \left(1-fct_{b}\right)\times \left(1-fct_{c}\right)\times \left(1-fct_{d}\right),\;1-fct_{e}\right\}\;$$

With agro-climatic constraints quantified, the agronomically attainable crop yields have been calculated by applying the factor (*fc*_3_) to the agro-climatic yields as calculated in Module II. Note that the evaluation is done separately for rain-fed and irrigated conditions.

**4. Module 4: Edaphic Assessment and Yield Reduction Due to Soil and Terrain Limitations**

The main purpose of Module IV is to provide for each crop/LUT a comprehensive soil suitability evaluation for all the soil units. This is done by the use of individual soil quality ratings (SQ). Seven different SQs are calculated and are combined in a soil unit suitability rating (SR, %). The SR represents the percentage of potential yield expected for a given crop/LUT with respect to the soil characteristics present in a soil map unit of the HWSD and is depending on input/management level.

Module IV produces a separate output file with soil evaluation results for each crop/LUT. A subset of information contained in these files is later on used in Module V when agro-ecological potential yields are estimated, accounting for yield reductions due to constraining soil and terrain-slope conditions. The output file is as a large matrix (in plain ASCII), with rows organized by soil map unit and individual component soil type and columns representing relevant soil unit characteristics followed by the estimated values of different soil qualities and the computed soil suitability ratings for each input/management level and terrain-slope class.

**5. Module 5: Integration of Results from Module 1–4 into Crop-Specific Grid-Cell Databases**

Module V executes the final step in the GAEZ crop suitability and land productivity assessment. It reads the LUT specific results of the agro-climatic evaluation for biomass and yield calculated in Module II/III for different soil classes and it uses the edaphic rating produced for each soil/slope combination in Module IV. The inventories of soil resources and terrain-slope conditions are integrated by ranking all soil types in each soil map unit with regard to occurrence in different slope classes. Considering simultaneously the slope class distribution of all grid cells belonging to a particular soil map unit results in an overall consistent distribution of soil-terrain slope combinations by individual soil association map units and 30 arc-sec grid cells. Soil evaluation and slope rules are applied separately for each water supply systems.

The algorithm in Module V steps through the grid cells of the spatial soil association layer of the Harmonized World Soil Database (HWSD) and determines for each grid cell the respective make-up of land units in terms of soil types and slope classes. Each of these component land units is separately assigned the appropriate suitability and yield values and results are accumulated for all elements. Processing of soil and slope distribution information takes place at 30 arc-second grid cells. One hundred of these produce the edaphic characterization at 5 arc-minutes, which is the resolution used for providing GAEZ results. As a result, information stored for 5 arc-minute grid cells contains distributions of the individual sub-grid evaluations.

The main purpose of Module V is to compile a grid-cell database for each crop or crop group storing evaluation results that summarize the processed sub-grid information. Computations include the following steps:

(1) Reading agro-climatic yields calculated for separate crop water balances of six broad soil AWC classes (from Module II/III);
(2) Applying AEZ rules for water-collecting sites (defined as Fluvisols and Gleysols on flat terrain);
(3) Applying reduction factors due to edaphic evaluation for the specific combinations of soil types/slope classes making up a grid-cell;
(4) Aggregating results over component land units (soil type/slope combinations), and calculating applicable fallow requirement factors depending on climate characteristics, soil type and crop group.

**6. Module 6: Actual Yield and Production**

To guide the spatial allocation of crops, GAEZ procedures for the calculation of potential yields and production have been applied to, respectively, rain-fed and irrigated cultivated land shares of individual 5 arc-minute grid-cells. Rather than taking an average yield for the entire grid cell it is assumed that the cultivated land will occupy the better of the suitability distribution determined in each grid cell. To estimate consistent spatial yield patterns of currently cultivated crops by grid-cells requires joint downscaling of agricultural statistics for all crops simultaneously. The sequential downscaling consists of efficient iterative rebalancing procedures based on cross entropy maximization principles, thereby allocating cropping activities to appropriate tracts of rain-fed respectively irrigated land while providing realistic estimates of current yield and production for the cultivated land in individual grid-cells, consistent with the land’s spatial distribution and agronomic capabilities.

In summary, two main steps were involved in obtaining downscaled grid-cell level area, yield and production of prevailing main crops:

(1) Estimation of shares of rain-fed or irrigated cultivated land by 5 arc-minute grid cell;
(2) Estimation of crop specific harvested area, yield and production of crops within the rain-fed and irrigated cultivated land of each grid cell.
